# Isolation, Identification, and Management Strategies for the Root Rot Pathogen of *Cardamine violifolia*

**DOI:** 10.3390/biology15040368

**Published:** 2026-02-22

**Authors:** Shaobing Gao, Wei Yang, Wenqin Bai, Yixuan Niu, Yalan Qiao, Yuchun Dai, Yutong Si, Xin Liu, Jie Xiang, Zhiwu Pei, Aimin Liang, Yuehua Xiao, Xin Cong, Jianyan Zeng

**Affiliations:** 1 College of Agronomy and Biotechnology, Southwest University, Chongqing 400715, China; 15238171793@163.com (S.G.); 15690636750@163.com (Y.N.); 15523540449@163.com (Y.Q.); 18290368483@163.com (Y.D.); 19112454844@163.com (Y.S.); 13140220296@163.com (X.L.); 13696145665@163.com (J.X.); 18790118962@163.com (Z.P.); liam2855@163.com (A.L.); xiaoyuehua@swu.edu.cn (Y.X.); 2 Enshi Se-Run Material Engineering Technology Co., Ltd., Enshi 445000, China; r41r32@163.com; 3 College of Horticulture and Gardening, Yangtze University, Jingzhou 434023, China; 4 Chongqing Academy of Agricultural Sciences, Chongqing 401329, China; bwqbc82@126.com; 5 National R&D Center for Se-Rich Agricultural Products Processing Technology, School of Modern Industry for Selenium Science and Engineering, Wuhan Polytechnic University, Wuhan 430048, China; 6 Hubei Enshi Selenium-Rich Resource Observation and Research Station, Wuhan 430048, China

**Keywords:** *Cardamine violifolia*, root rot, fungicide synergism, fungicide screening, cell membrane disruption

## Abstract

Root rot disease severely impacts the yield of *Cardamine violifolia*, a selenium-enriched cruciferous vegetable. However, the causal pathogens and effective control strategies of this disease remain poorly characterized. This study systematically isolated and identified three key pathogens from diseased tissues in the Enshi region: *Aspergillus costaricensis*, *Mucor circinelloides* cf. *lusitanicus*, and *Fusarium pernambucanum*. Morphological characterization, phylogenetic analysis, and pathogenicity testing were conducted. Candidate fungicides were screened using plate inhibition assays, and combinations were optimized and validated through soil drenching experiments. While propiconazole showed broad-spectrum activity, its efficacy against *Aspergillus* and *Mucor* was suboptimal. A novel ternary compound fungicide, T10, combining propiconazole, hymexazol, and difenoconazole, demonstrated significantly enhanced potency with EC_50_ values of 7.313, 12.2983, and 0.1781 mg/L against the three pathogens, representing reductions of 66.0%, 77.7%, and 92.1% compared to the most effective single application of propiconazole. At 10 mg/L, T10 increased inhibition rates by 62.62%, 77.53%, and 20.85% against the three pathogens, respectively, compared to propiconazole alone. Propidium iodide (PI) staining revealed increased cell membrane permeability in T10-treated pathogens, suggesting that membrane damage may contribute to its antifungal effect. This study provides a robust scientific basis for sustainable disease management of this high-value selenium-enriched vegetable crop.

## Simple Summary

This study not only confirms the existence of key pathogens of *Cardamine violifolia* root rot but also proposes and validates an efficient chemical control strategy centered on the propiconazole–hymexazol–difenoconazole formulation. This strategy provides a theoretical foundation and technical support for the healthy production of this plant, while also offering new research directions and practical models for comprehensive and efficient control of root rot in Brassicaceae crops.

## 1. Introduction

*Cardamine* spp. belong to the Brassicaceae family and are a traditional medicinal and edible plant resource in southwestern China. Among these species, *C. violifolia* has attracted widespread attention due to its exceptional selenium-enrichment capacity and unique nutritional value. As a typical hyper-selenium-enriching plant discovered in Enshi, Hubei Province, its aboveground selenium content can exceed 1000 mg/kg under wild conditions and 9000 mg/kg under artificial selenium-enriched cultivation conditions [1]. Studies have shown that extracted selenium proteins and peptides of *C. violifolia* have high bioavailability, with organic selenium content significantly higher than that of ordinary selenium-enriched plants [2]. In recent years, as the biological functions of selenium in immune regulation, antioxidation, and neuroprotection have been increasingly validated, the application value of *C. violifolia* in the functional vegetable and health food industries has continued to rise [3]. Nervonic acid, a crucial lipid for myelin sheath formation and nervous system development, has been recognized in recent nutritional medicine research for its potential role in enhancing cognitive function and delaying the progression of neurodegenerative diseases [4]. Studies show that the nervonic acid content in the seed oil of some *Cardamine* species approaches 50% of total fatty acids, providing an important direction for developing new functional oil crops [5,6]. Currently, *C. violifolia* has achieved large-scale cultivation in Enshi and other regions and has received approval from China’s National Health Commission as a new food raw material. As such, it is widely used in the development of selenium-enriched vegetables, functional foods, and organic selenium extracts. Furthermore, the selenium-enrichment characteristics of *C. violifolia* provide ideal material for elucidating the mechanisms of selenium uptake, transport, and metabolic regulation in plants [7]. Moreover, the complete genome sequencing of *C. violifolia* and *Cardamine hupingshanensis* provides key genetic resources for dissecting hyper-selenium-enrichment traits [7,8]. Therefore, *Cardamine* holds significant value from the perspectives of nutritional health, agricultural industrialization, and plant functional gene research, and its resource development has become a focal point in agricultural and functional plant research.

Root rot is one of the most common and severely damaging soil-borne diseases in crop production, primarily caused by complex infection of various pathogenic fungi in soil, including *Fusarium* spp., *Rhizoctonia solani*, *Phoma* spp., and *Aphanomyces euteiches* [9]. These pathogens generally use soil or plant residues as their primary sources of infection. They invade through root epidermis or wounds, leading to vascular tissue necrosis, root rot, aboveground wilting, and even plant death. Root rot is characterized by strong concealment, long incubation periods, and diverse transmission pathways [9]. Moreover, environmental conditions such as high temperature and humidity, poor drainage, and continuous cropping significantly increase the occurrence and spread rate of root rot [10,11]. Root rot has caused significant losses in various economic crops. For example, sweet potato root rot under high-temperature and drought conditions can cause a yield loss of 20–80%, and even total crop failure in extreme cases [12]. In Brassicaceae vegetable production, root rot often causes large-scale yield reductions. For instance, in Canadian production areas of *Brassica napus*, yield loss in severely affected plots can reach 5–35%, with near-total loss under serious conditions [13]. Given the taxonomic status and similarities in cultivation between *Cardamine* and Brassicaceae vegetables, as well as its production in moist (approximately 28 °C, humidity > 60%), nutrient-rich environments, which are the key conditions for high root rot incidence, the disease risk for *Cardamine* is increased. In recent years, with the rapid expansion of the *C. violifolia* industry, root rot has become a key factor restricting its production. Once infected during the seedling stage or early transplanting period, it often leads to reduced survival rates, yield decline, and increased cultivation management costs. Despite the increasing cultivation scale and economic value of *C. violifolia*, the causal pathogens of its root rot disease remain to be systematically characterized, hindering the development of effective disease management strategies. Therefore, clarifying the main root rot pathogens, understanding disease occurrence patterns, and conducting effective control research are prerequisites for the sustainable development and industrial expansion of *Cardamine*.

Although agricultural measures such as crop rotation, improved drainage, and soil sterilization can reduce the occurrence of root rot to some extent, chemical fungicides are one of the most direct and effective measures in current control systems [14]. Current chemical agents targeting major root rot pathogens (e.g., *Fusarium oxysporum*, *Rhizoctonia solani*, and *Phoma* spp.) include benzimidazole fungicides (carbendazim and thiophanate-methyl), phenylpyrrole fungicides (fludioxonil and difenoconazole), triazole fungicides (propiconazole and difenoconazole), and fluoro-pyridines (fluazinam). Among these agents, carbendazim exhibits significant efficacy against *Fusarium* root rot by inhibiting tubulin and blocking fungal cell division [15]. Hymexazol is a systemic fungicide with both soil disinfection and growth-promoting effects, primarily used to control root rot and soil-borne diseases in rice and various vegetables caused by *Fusarium*, *Pythium*, and *Aphanomyces* [16,17]. Triazole demethylation inhibitor (DMI) fungicides, such as propiconazole and difenoconazole, can inhibit ergosterol biosynthesis in fungal cell membranes, damaging pathogen membrane structure and inhibiting expansion, exhibiting good efficacy against root rot caused by *Rhizoctonia solani* [18]. Although nearly 3.5 million tons of synthetic pesticides are used each year for disease control, less than 0.1% reach their targets, while over 99% fail to exert their intended effects [19]. Long-term reliance on single-chemical fungicides has led to a progressive increase in pathogen resistance. For example, 55 isolates of *Fusarium graminearum* from Belgium, Canada, the USA, Germany, Italy, and Luxembourg between 1969 and 2009 had intrinsic resistance to trifloxystrobin [20]. Developing appropriate pesticide delivery systems and efficient, environmentally friendly pesticide formulations to reduce pesticide usage is a current focus of research. Researchers are currently developing fungicide formulations and new targets for fungicide discovery. For instance, the combination of flutriafol and propiconazole showed excellent efficacy in vitro against wheat *Fusarium* crown rot [21]. By contrast, novel isoxazoline-class agents with a pyrazole–piperidine–thiazole–isoxazoline heterocyclic skeleton exhibit high activity against oomycete diseases, such as Phytophthora and Pythium, by targeting oxysterol-binding proteins (OSBPs) [22]. Additionally, some modern fungicides can induce plant defense responses and enhance host resistance under low-dose conditions. Research has found that the combined application of low-dose tebuconazole·azoxystrobin and tetramycin can improve resistance, growth, and quality of *Pseudostellaria heterophylla* against leaf spot and viral diseases [23].

To address the challenges of controlling root rot of *C. violifolia*, this study utilized infected seedlings from Enshi, Hubei Province, as experimental material. Through tissue isolation, morphological observation, and molecular identification, we aimed to clarify the primary pathogenic species responsible for *C. violifolia* root rot. Additionally, through primary and secondary screening of commonly used fungicides, we identified and optimized formulations with significant inhibitory effects against these pathogens, and further explored their antimicrobial mechanisms. Overall, this study not only elucidates the primary pathogenic spectrum and characteristics of *C. violifolia* root rot but also proposes an efficient and environmentally friendly control strategy based on fungicide combinations. These findings provide a theoretical foundation and technical support for the sustainable development of the *C. violifolia* industry, while also offering novel insights into the precision management of root rot in Brassicaceae plants.

## 2. Materials and Methods

### 2.1. Isolation, Purification, and Preservation of Root Rot Pathogens

**Figure 1:**
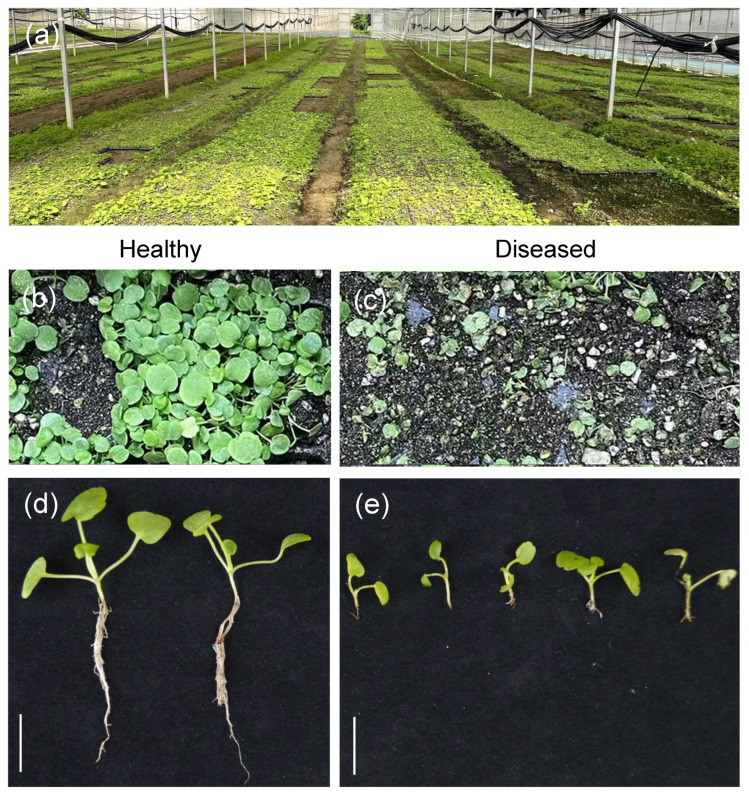
Symptoms of root rot in *C. violifolia*. (**a**) Severe disruption of the seedling cultivation process by root rot at the *C. violifolia* nursery base of Enshi Se-Run Material Engineering Technology Co., Ltd. in 2024. (**b**,**d**) Healthy *C. violifolia* seedlings; (**c**,**e**) *C. violifolia* seedlings infected with root rot. Scale bar = 1 cm.

Root rot pathogens were isolated using the conventional tissue isolation method described by Sahu et al. [24] and Jhuma et al. [25] with appropriate modifications. Root rot-infected plants were collected in October 2024 from the *C. violifolia* nursery base of Enshi Se-Run Material Engineering Technology Co., Ltd., Enshi, Hubei Province, China. Plants were randomly selected from infected areas (Figure 1a), and disease symptoms were documented photographically before laboratory processing. Samples were first rinsed with clean water to remove surface soil, and then approximately 1 cm long tissue sections at the disease–healthy interface were excised. In a sterile laminar flow hood, the tissues were sequentially soaked in 75% ethanol for 30 s, sterilized with 10% hydrogen peroxide for 30 s, and rinsed three times with sterile water. Subsequently, moisture on the root surface was absorbed with sterile filter paper; the diseased portions were cut open with a sterile scalpel; and the cut sections were placed on Potato Dextrose Agar (PDA) medium (46 g/L, Coolaber, Beijing, China) supplemented with cephalosporin (concentration 0.1%), followed by upside-down incubation in an incubator at 28 ± 0.5 °C for 5–7 d. When colonies of different morphology grew from the root tissue onto the plates, purification was performed. Hyphae from the edges of colonies with various morphologies were extracted using a sterile inoculation needle and transferred to new medium, followed by culture in a constant temperature incubator at 28 ± 0.5 °C. After three successive subcultures, some strains were used for experiments, while others were preserved on PDA slants (4 °C) and in 20% glycerol (−60 °C) [26]. To verify that the isolated strains fulfilled Koch’s postulates, pathogenicity tests were performed on the primary pathogenic strains A2, A3, and A6 as follows: (1) consistent isolation from diseased root tissues; (2) pure culture establishment; (3) reproduction of typical root rot symptoms upon inoculation of healthy seedlings; and (4) successful re-isolation from symptomatic tissues and confirmation of strain identity to the original inocula.

### 2.2. Observation of Pathogen Morphology

The isolated and purified pathogen was extracted with a sterile pipette tip (φ = 6 mm) and re-inoculated onto fresh PDA medium (46 g/L) as mycelial plugs. After incubation at 28 ± 0.5 °C for 7 days, the growth and morphological characteristics of hyphae and spores were examined using an upright fluorescence microscope DMRXA2 (Leica, Wetzlar, Germany) and a scanning electron microscope (SEM, Hitachi, Tokyo, Japan) at an accelerating voltage of 2 kV [27].

### 2.3. Molecular Biological Identification of Pathogens

Genomic DNA was extracted from the strains using the CTAB method [28] with appropriate modifications and stored at −20 °C. The internal transcribed spacer (ITS) region of rDNA (rDNA-ITS) was amplified using the universal fungal identification primers ITS1 (5′-TCCGTAGGTGAACCTGCGG-3′) and ITS4 (5′-TCCTCCGCTTATTGATATGC-3′) [29]. PCR cycling conditions were as follows: initial denaturation at 94 °C for 5 min; 35 cycles of denaturation at 94 °C for 30 s, followed by annealing at 48 °C for 40 s, and extension at 72 °C for 1 min; and a final extension at 72 °C for 10 min. The RNA polymerase II second-largest subunit (RPB2) region was amplified using primers fRPB2-5f (5′-GAYGAYMGWGATCAYTTYGG-3′) and fRPB2-7cR (5′-CCCATRGCTTGYTTRCCCAT-3′) [9]. PCR cycling conditions were as follows: initial denaturation at 95 °C for 5 min; 35 cycles of denaturation at 95 °C for 1 min, followed by annealing at 55 °C for 90 s, and extension at 72 °C for 90 s; and a final extension at 72 °C for 7 min. The amplification products were sent to Sangon Biotech (Shanghai, China) Co., Ltd. for sequencing. The obtained gene sequences were submitted to the National Microbiology Data Center website (NMDC, http://nmdc.cn/, accessed on 21 October 2024) to obtain accession numbers. BLAST analysis was performed on the NCBI website (https://www.ncbi.nlm.nih.gov/, accessed on 19 March 2025) for sequence comparison, and sequences with >95% similarity were downloaded (Tables S1 and S2). Subsequently, the sequences rDNA ITS and RPB2 were concatenated end-to-end [30], and a phylogenetic tree was constructed using the Neighbor-Joining method in MEGA12 (v. 12.0.12, Temple University, Philadelphia, PA, USA) [31].

### 2.4. Pathogenicity Test

The pathogenicity test was conducted using a mycelial plug wound inoculation method [32] with appropriate modifications.

For the mycelial plug inoculation experiment, the preserved purified strains were reactivated and cultured on PDA medium (Coolaber, Beijing, China) at 28 ± 0.5 °C in darkness for 7 days. Healthy *C. violifolia* seedlings grown for 7 days after germination were selected, and the stem base of each seedling was wounded using a sterile toothpick (2 wound sites per plant). Mycelial plugs were obtained using a sterile pipette tip (φ = 6 mm) and transferred to the wound sites with a sterile toothpick. The inoculated areas were wrapped with moistened absorbent cotton and covered with adhesive plastic wrap to maintain humidity. Each fungal treatment group contained three biological replicates. Plants inoculated with sterile PDA plugs served as the negative control, and moisture was maintained using the same method as the treatment groups. Disease development in both treatment and control groups was observed and recorded 14 days post-inoculation (dpi).

The soil drench inoculation experiment was similar to the mycelial plug inoculation experiment. Healthy *C. violifolia* seedlings grown for 7 days after germination were selected, and the stem base was wounded with a sterile toothpick (2 wound sites per plant). The preserved purified strains were reactivated and cultured on PDA medium (Coolaber, Beijing, China) at 28 ± 0.5 °C in darkness for 48 h. Single colonies were inoculated into 50 mL of Potato Dextrose Broth ( PDB) medium and shake-cultured at 28 °C and 160 r/min for 48 h. The culture broth was centrifuged at 4000 r/min for 15 min. The precipitated cells were then collected and diluted with sterile water to prepare a spore suspension of 10^6^ CFU/mL. Each pot received 10 mL of the spore suspension as a soil drench. Disease investigation was conducted after 7 or 14 days of cultivation by washing the roots with sterile water. Pots were drenched with an equal volume of deionized water, serving as the negative control [33].

### 2.5. Screening of Fungicides and Inhibition Tests

For the preliminary screening of fungicides, eight agents that were previously reported to be widely used in root rot control were selected for toxicity testing against the most pathogenic strain A6: hymexazol (T1), difenoconazole (T2), prochloraz (T3), metalaxyl/mancozeb (T4), chlorothalonil (T5), carbendazim (T6), kasugamycin/oxine–copper (T8), and propiconazole (T9) (Table S3). Based on the active ingredients of each fungicide, the corresponding mass was accurately weighed and dissolved in sterile water to prepare a stock solution of 5000 mg/L. Subsequently, 0.5 mL of the stock solution was added to 49.5 mL of PDA medium (46 g/L) to formulate fungicide-amended media at a concentration of 50 mg/L. In contrast, PDA medium without fungicide served as the negative control. Each treatment was performed with three biological replicates. Using a sterile pipette tip (φ = 6 mm), plugs were taken from the edge of 5-day-old colonies and inoculated onto the center of each medium plate. After incubation at 28 °C in darkness for 5 days, the colony diameters (including the initial inoculated mycelial plug of 6 mm) were measured using the cross method, and the inhibition rates for each fungicide concentration against mycelial growth were calculated. The inhibition rate (%) was calculated using formula (1): [(Negative control colony diameter − Treatment colony diameter)/(Negative control colony diameter)] × 100% [34,35].

For the secondary screening of fungicides, agents showing >30% inhibition in the preliminary screening tests were selected for further testing against strains A6, A2, and A3. These fungicides were prepared at a series of concentrations: the first at 50 mg/L, the second at 40 mg/L, the third at 30 mg/L, the fourth at 20 mg/L, the fifth at 10 mg/L, and lower concentrations for PDA test plates with T9 treatment (e.g., 0.625 mg/L, 1.25 mg/L, 2.5 mg/L, and 5 mg/L). Using the mycelial plug preparation method described above, we prepared 6 mm diameter plugs that were inoculated at the center of each plate and incubated at 28 ± 0.5 °C in darkness for 5 days, with colony diameters measured and inhibition rates calculated using the same methods as the preliminary screening. Regular PDA plates without fungicide served as the negative control. The experiment consisted of five treatments with three replicates per treatment. Measures included the inhibition rate (Y) and the logarithm of fungicide concentration (X), the toxicity regression equation (Y = aX + b), correlation coefficient (r), and median effective concentration (EC_50_, mg/L).

### 2.6. Preparation of Compound Fungicides and Their Inhibition Tests

Fungicides with an inhibition rate of more than 30% were selected and combined as a novel compound fungicide, T10. The components of this compound included propiconazole, hymexazol, and difenoconazole.

Specific formulations for the compound fungicide in plate tests were as follows: When using strains A2 and A3 as pathogens, the concentration gradients of hymexazol (T1), difenoconazole (T2), and propiconazole (T9) in the compound fungicide were 10 mg/L, 20 mg/L, 30 mg/L, 40 mg/L, and 50 mg/L. When using strain A6 as the pathogen, low-concentration gradient hymexazol (T1), difenoconazole (T2), and propiconazole (T9) were applied to the compound fungicide at 0.625 mg/L, 1.25 mg/L, 2.5 mg/L, and 5 mg/L, respectively. Using Excel and the DPS data processing system, the toxicity regression equations, median effective concentrations (EC_50_ values), and correlation coefficients (r) were calculated for each fungicide.

Specific formulations for each fungicide in soil condition tests were as follows: individual application of hymexazol (T1) at a target concentration of 500 mg/L; individual application of difenoconazole (T2) at 100 mg/L; individual application of propiconazole (T9) at 250 mg/L; and combined application of the three-compound agent (T10). Respective target concentrations of 120 mg/L were used for each fungicide. Controls: Negative controls included PDA plates without fungicide. Propiconazole (T9) at 50 mg/L, which showed the best fungicidal efficacy, served as the positive control.

Synergism analysis of the T10 compound fungicide was performed using Wadley’s method [36]. The theoretical half-maximal effective concentration, EC_50_(th), was calculated based on the three individual fungicides (T1, T2, and T9) using the formula (2): EC_50_(th) = (a + b + c)/[a/EC_50_(A) + b/EC_50_(B) + c/EC_50_(C)], where A, B, and C represent the three fungicides, and a, b, and c represent their respective proportions in the mixture. The experimentally observed EC_50_, EC_50_(ob), was determined from the actual bioassay. The synergism ratio (SR) was calculated using the formula (3): SR = EC_50_(th)/EC_50_(ob). An SR > 1.5 indicates synergism; SR = 0.5–1.5 indicates additive effects; and SR < 0.5 indicates antagonism.

### 2.7. Determining Efficacy of Fungicides in Root Rot Disease Control

First, humus soil was sterilized by autoclaving at 100 °C until dry; then, it was fully moistened with sterile water. Germinated *C. violifolia* seeds were sown in the soil and cultivated at 22 °C with a photoperiod of 16 h light/8 h dark. Healthy *C. violifolia* seedlings were selected at 7 days post-germination, and the stem base of each plant was wounded at two points with a sterile toothpick. The isolated pathogens (strains A2, A3, and A6 in this study) were cultured on PDA medium at 28 °C for 48 h. Mycelial mats were scraped and inoculated into 50 mL of PDB medium and then shaken at 28 °C and 160 rpm for 48 h. After filtration through gauze, a spore suspension of 1 × 10^6^ CFU/mL was prepared with sterile water. Each nursery pot was drenched with 10 mL of spore suspension for each pathogen and placed in the dark for 24 h. Three days after pathogen inoculation, 10 mL of fungicide (single–agent or compound formulation) was applied to each nursery pot via root drenching to stimulate rot. Root drenching with an equal volume of sterile water served as the negative control. At 7 days post-treatment, disease development was assessed. Disease index = [Sum (Disease score × Number of plants at that grade)/(Total number of surveyed plants × Highest grade value)] × 100. The grading scale for root rot in *C. violifolia* seedlings was based on the method of Jiao et al. [37] with slight modifications: Grade 0: green leaves, healthy root and stem development with no lesions; Grade 1: slight root discoloration and browning area < 10% of total root area; Grade 2: partial root browning wih 10–30% browning; Grade 3: roots segmentally brown to black, constricted and dry, lateral roots partially broken, and leaves chlorotic and brown, with 30–50% browning; and Grade 4: roots segmentally brown to black, constricted, >50% browning, lateral roots broken, and leaves are yellow, near-death or dead.

### 2.8. PI Fluorescent Staining Analysis

Detection of cell membrane permeability in root rot pathogen hyphae was performed based on the propidium iodide (PI) staining method used by Rosenberg et al. [38] with slight modifications. Hyphae from the colony edge grown on PDA plates for 5 days after treatment with different fungicide concentrations were selected, and the mycelia were washed three times with phosphate-buffered saline (PBS, 0.1 mol/L, pH = 7.0). The washed mycelia were transferred to clean glass slides; then, 50 μL of PI dye (1 mg/mL) was added and stained for 30 min in the dark. After staining, excess PI dye was washed away with PBS again; a cover slip was placed; and the PI staining results of hyphae were observed using an inverted fluorescence microscope BX81 (Olympus, Tokyo, Japan). Strains treated with PBS alone served as negative controls.

### 2.9. Data Analysis and Statistics

The colony diameter (D) of each treatment was measured using the cross method, and the inhibition rates of different fungicide concentrations on mycelial growth were calculated. Probability values of inhibition rates were plotted as the ordinate (Y) against logarithmic fungicide concentrations as the abscissa (X) to generate toxicity regression equations (Y = aX + b), correlation coefficients (r), and median effective concentrations (EC_50_, mg/L). All experiments were performed with three independent biological replicates, with data presented as mean ± standard deviation (SD). Data processing was conducted using MS Excel 2019 (Microsoft, WA, USA) and the DPS data processing system (v. 7.05, Hangzhou RuiFeng Information Technology, Hangzhou, China). Statistical analyses and graphing were performed with GraphPad Prism 10 (GraphPad Software, CA, USA), including one-way ANOVA with Tukey’s HSD post hoc test for multiple comparisons of fungicide efficacy, and Pearson correlation analysis for concentration–response relationships. Statistical significance was set at *p* < 0.05. Critical values for Pearson r with df = 3 (*n* = 5 concentration gradients) were r_0.05_ = 0.878 and r_0.01_ = 0.959.

## 3. Results

### 3.1. Root Rot Symptoms and Isolation of Pathogens in C. violifolia

Diseased *C. violifolia* seedlings exhibiting root rot symptoms were collected from the nursery base of Enshi Se-Run Material Engineering Technology Co., Ltd. (Enshi, China) (Figure 1a) for morphological characterization. The results showed that in the early stage of disease onset, young roots typically presented a water-soaked, brownish discoloration. The root epidermal layer and xylem gradually turned black as the pathogen continuously spread upward along the root tissue. As the disease progressed further, the root epidermal layer showed soft rot, the vascular bundles became blocked and blackened, the root system rotted and disintegrated, and the above-ground leaves began to yellow. In severe cases, entire plants wilted and turned yellow-brown until death (Figure 1b–e).

### 3.2. Morphological Observation and Molecular Identification of Isolated Pathogens

**Figure 2:**
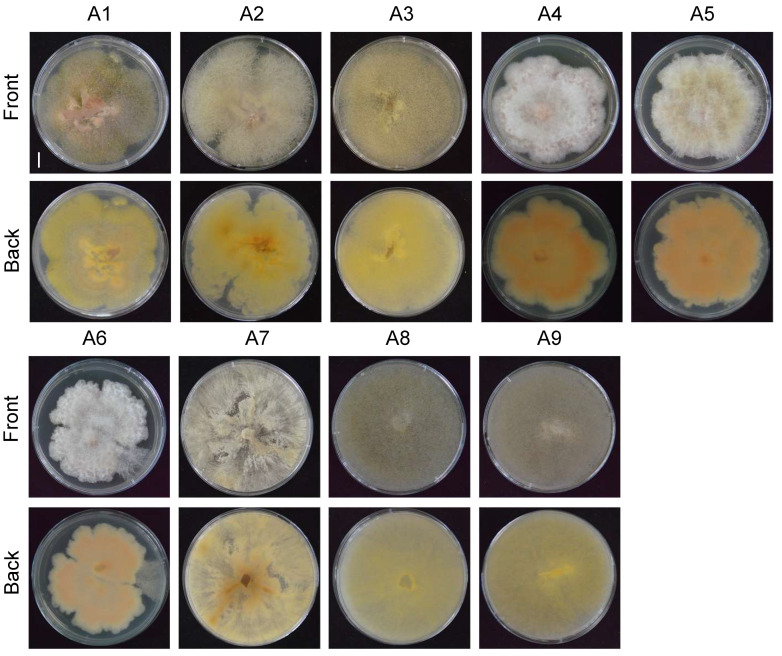
Morphological differences in nine colonies isolated from the roots of diseased plants on plates. Colony morphology after 12 days of incubation. Front, front view; Back, back view. (A1–A9): designations for nine different strains isolated from diseased plants. Scale bar = 1 cm.

**Figure 3:**
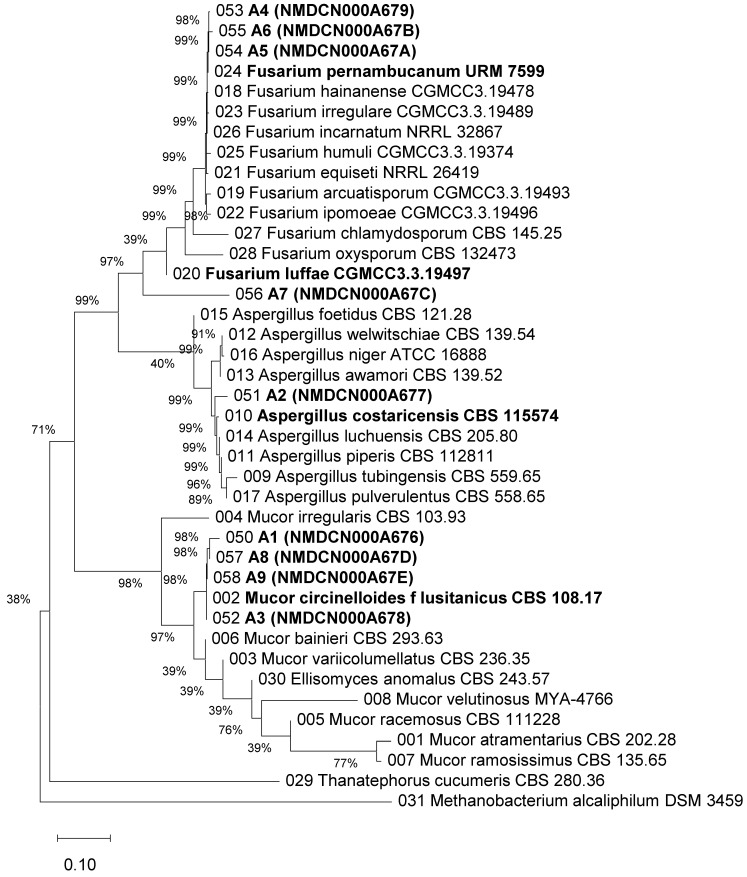
Phylogenetic tree constructed based on concatenated rDNA ITS and RPB2 sequences. (A1–A9): designations for nine different strains isolated from diseased plants. Genomic DNA was extracted from all tested strains. The rDNA internal transcribed spacer (ITS) region and RNA polymerase II second largest subunit (RPB2) gene were amplified using primer pairs ITS1/ITS4 and fRPB2-5f/fRPB2-7cR, respectively. The obtained sequences were submitted to the National Microbiology Data Center (NMDC, http://nmdc.cn/) for accession numbers and subjected to BLASTn analysis against the NCBI nucleotide database (https://www.ncbi.nlm.nih.gov/). Reference sequences exhibiting > 95% similarity were retrieved for subsequent analysis. The ITS and RPB2 sequences were concatenated, and phylogenetic reconstruction was performed using the Neighbor-Joining algorithm implemented in MEGA12 software.

The pathogens were isolated from the infected parts of plants with root rot disease using the conventional tissue isolation method [24]. By purifying colonies with different morphologies, nine strains with distinct colony morphologies and colors were obtained and designated as A1–A9 (Figure 2). To identify the pathogen species, genomic DNA was extracted from each strain, and the universal fungal identification primer pairs ITS1 + ITS4 and fRPB2-5f + fRPB2-7cR were used to amplify the rDNA ITS and RPB2 sequences, respectively. The sequences rDNA ITS and RPB2 were concatenated end-to-end, and a phylogenetic tree was constructed using the Neighbor-Joining method in MEGA12 software (Figure 3).

Based on the phylogenetic clustering and sequence homology analysis results, strains A1, A3, A8, and A9 clustered with *Mucor circinelloides* cf. *lusitanicus* (Figure 3). Their coverages against the reference sequence (GenBank accession number: CBS 108.17) were 100%, 100%, 100%, and 99%, respectively, with similarities of 98.03%, 99.88%, 99.54%, and 99.88% (Table S2). Strain A2 clustered with *Aspergillus costaricensis* (Figure 3), showing 100% coverage and 96.55% similarity to the reference sequence (GenBank accession number: CBS 115574) (Table S2). Strains A4, A5, and A6 clustered with *Fusarium pernambucanum* (Figure 3) and had 100% coverage against the reference sequence (GenBank accession number: URM 7599) and similarities of 99.54%, 96.54%, and 96.54%, respectively (Table S2). Strain A7 clustered with *Fusarium luffae* (Figure 3), with 98% coverage and 95.87% similarity to the reference sequence (GenBank accession number: CGMCC3.3.19497) (Table S2).

### 3.3. Strains A2, A3, and A6 Are the Main Pathogens of C. violifolia Root Rot

**Figure 4:**
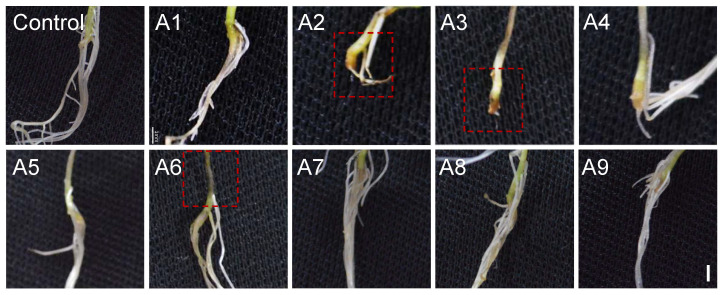
Pathogenicity assessment of isolates from *C. violifolia* root rot using the mycelial plug wound inoculation method. Symptom development of *C. violifolia* in the stem base at 14 days post-inoculation. Red dashed boxes indicate the disease sites. Scale bar = 1 mm.

**Figure 5:**
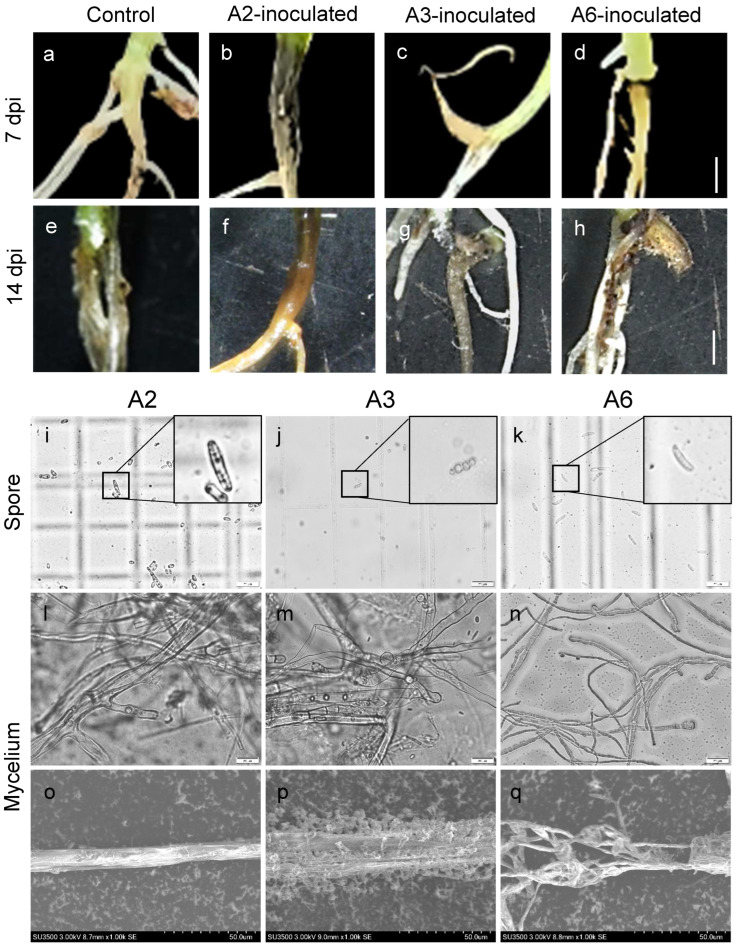
Pathogenicity assessment of strains A2, A3, and A6 of *C. violifolia* root rot using root-drench inoculation under soil cultivation conditions, and observation of hyphal and spore morphologies. (**a**–**h**) Disease symptoms of *C. violifolia* in the stem base 7 and 14 days post-inoculation. Scale bar = 2 mm. (**i**–**n**) Morphology of conidia (**i**–**k**) and hyphae (**l**–**n**) in the strains observed under an inverted microscope. Images in the upper right corner show enlarged views of the boxed areas. Scale bar = 20 μm. (**o**–**q**) Hyphal morphology of the strains observed by scanning electron microscopy. Scale bar = 50 μm.

After 14 days of inoculation with strains A2, A3, and A6 using an improved mycelial plug wound inoculation method, blackening of the stem base and root breakage occurred in *C. violifolia*, while no obvious disease symptoms were observed with other strains or the negative control, indicating that strains A2, A3, and A6 are the main pathogens causing root rot in this species (Figure 4). Under soil cultivation conditions, the pathogenicity of strains A2, A3, and A6 was further verified by drenching the roots of robust *C. violifolia* seedlings at 7 days post-germination with a spore suspension of 10^6^ CFU/mL. After 7 days of root drenching, obvious blackening of the seedling neck region was observed, and after 14 days, the symptoms further intensified with root rot and breakage (Figure 5a–h). Based on these results, three strains were identified as the main pathogens of *C. violifolia* root rot. Furthermore, the hyphal and spore morphologies of strains A3, A4, and A6 were observed under an inverted fluorescence microscope and a scanning electron microscope. The results showed that pathogen A2 had a velvety mycelial texture with conidia arranged in chains, similar to that of *Aspergillus*; pathogen A3 had a cottony mycelial texture with conidia arranged in grape-like clusters, a morphology similar to *Mucor*; and pathogen A6 had white fluffy mycelium with falcate conidia, consistent with the morphology of *Fusarium* (Figure 5i–q). Furthermore, re-isolation of fungal strains from symptomatic root tissues yielded colonies with morphological characteristics identical to those of the original inoculated strains A2, A3, and A6 (Figure S1), thereby strictly fulfilling Koch’s fourth postulate and confirming the pathogenic etiology of these isolates.

### 3.4. Fungicides T1, T2, and T9 Significantly Inhibit the Growth of the C. violifolia Root Rot Pathogen

**Figure 6:**
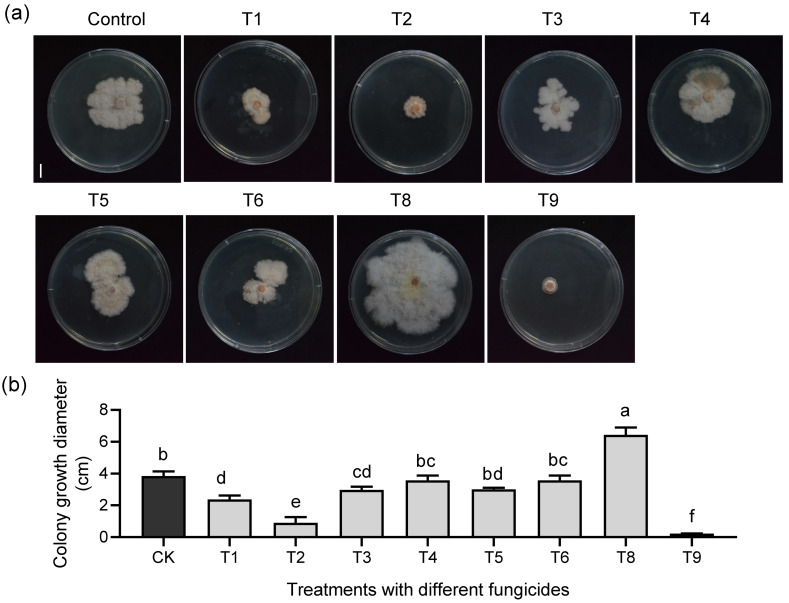
Effects of different fungicides on the growth rate of the A6 pathogen causing *C. violifolia* root rot. (**a**) Colony growth on medium supplemented with 50 mg/L of different fungicides after 5 days of incubation. Colonies grown on fungicide-free medium served as the negative control. Scale bar = 1 cm. T1–T9: designations for eight fungicide treatments, namely hymexazol (T1), difenoconazole (T2), prochloraz (T3), metalaxyl–mancozeb (T4), chlorothalonil (T5), carbendazim (T6), kasugamycin·oxine–copper (T8), and propiconazole (T9). (**b**) Statistical results of colony diameter for A6 pathogen after 5 days of treatment with different fungicides (diameter includes the initial inoculated mycelial plug of 6 mm). Error bars indicate the standard deviation (SD) of three plates in each experiment. Different letters in (**b**) represent significant differences at *p* < 0.05 using one-way ANOVA with Tukey’s multiple comparisons test.

Given that *Fusarium* spp. are primary causal agents of root rot and strain A6 exhibited strong pathogenicity against *C. violifolia*, the mycelial growth rate method was employed using this strain to preliminarily assess the toxicity of eight fungicides widely reported for root rot control: hymexazol (T1), difenoconazole (T2), prochloraz (T3), metalaxyl–mancozeb (T4), chlorothalonil (T5), carbendazim (T6), kasugamycin/oxine–copper (T8), and propiconazole (T9) (Table S3). After 5 days of incubation with 6 mm mycelial plugs at 50 mg/L, colony diameters on media containing T1, T2, T3, T8, and T9 differed significantly from the negative control, whereas other treatments showed no significant differences. Notably, T9 exhibited the strongest inhibitory effect (93.99% inhibition), while T8 promoted pathogen growth (−68% inhibition); the underlying mechanism remains unclear (Figure 6; Table S4). Fungicides T1, T2, and T9, which achieved inhibition rates exceeding 30%, were subsequently selected for secondary screening against pathogens A2, A3, and A6 (Figure 6; Table S4).

**Figure 7:**
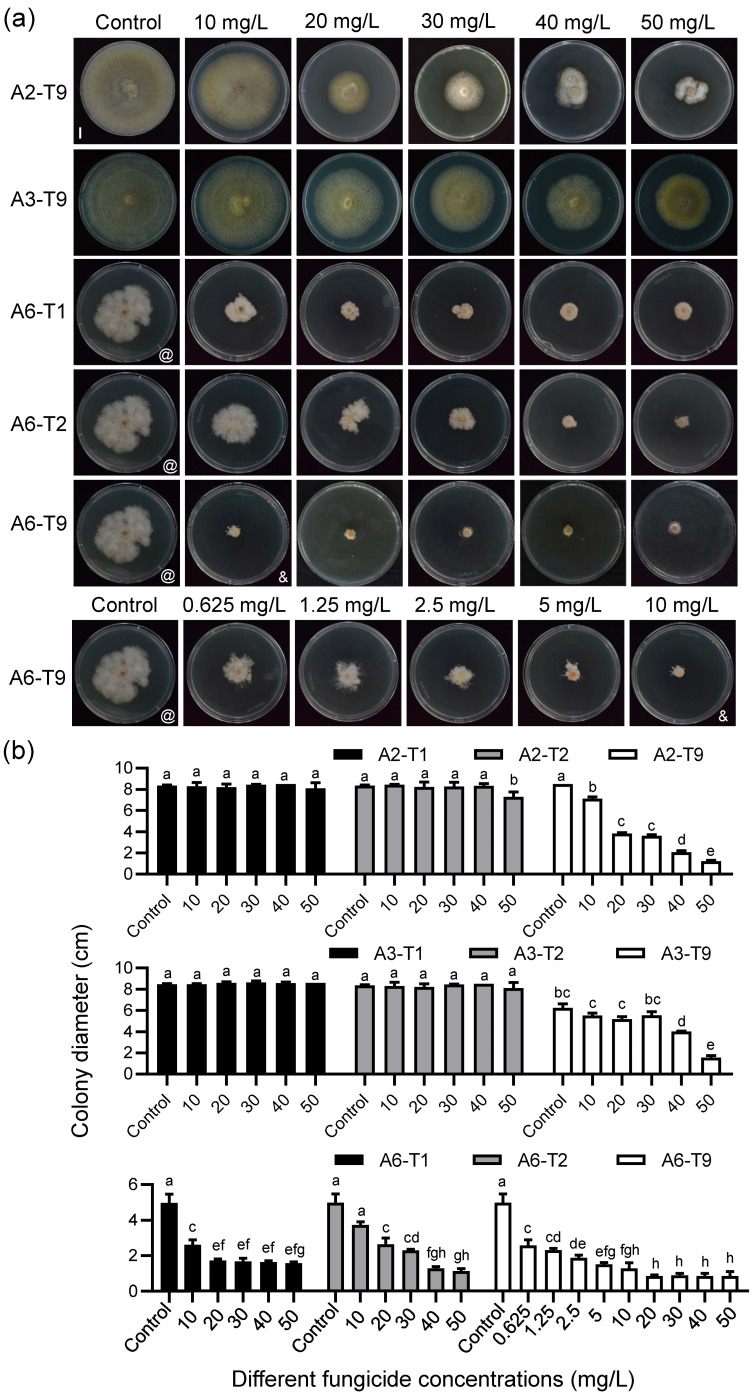
Effects of different concentrations of T1, T2, and T9 fungicides on the growth rate of pathogenic strains A2, A3, and A6 for *C. violifolia* root rot. (**a**) Colony growth of strains A2, A3, and A6 after 5 days of incubation on media containing different concentrations of fungicides T1, T2, and T9. Colonies grown on fungicide-free medium served as the negative control. Scale bar = 1 cm. Symbols @ and & indicate results for the same strain under the same treatment condition. (**b**) Statistical results of colony diameters for pathogenic strains A2, A3, and A6 after 5 days of incubation on media containing different concentrations of fungicides T1, T2, and T9 (the diameter includes the initial inoculated mycelial plug of 6 mm). Error bars indicate the SD of three plates in each experiment. Different letters in (**b**) represent significant differences at *p* < 0.05 using one-way ANOVA with Tukey’s multiple comparisons test.

Furthermore, to test the toxicity of the fungicides initially against A2, A3, and A6 *C. violifolia* pathogens, different concentrations of T1, T2, and T9 were used. Among the three tested fungicides, T9 (propiconazole) demonstrated a degree of inhibition against the three pathogens, with rates of 74.42%, 43.11%, and 92.04% observed against A2, A3, and A6 at a concentration of 50 mg/L, respectively (Figure 7; Table S5). The EC_50_ values for A2, A3, and A6 were 21.5214 mg/L, 55.0963 mg/L, and 2.2504 mg/L, respectively (Table 1). However, the EC_50_ values for A2 and A3 were greater than 20 mg/L, and the inhibition rate for A3 was less than 50%, indicating a poor inhibitory effect (Table 1). Additionally, T1 and T2 fungicides had inhibition rates of 78.11% and 88.24% against A6 at a concentration of 50 mg/L, respectively (Figure 7; Table S6). Both had EC_50_ values less than 20 mg/L, indicating good inhibitory effects against A6 (Table 1). However, different concentrations of T1 and T2 fungicides showed no significant inhibitory effect on A2 and A3 (Figure 7).

These results demonstrated that fungicides T1, T2, and T9 exhibit significant inhibitory activity against *C. violifolia* root rot pathogens, particularly pathogen A6. However, their efficacy against pathogens A2 and A3 was less pronounced and requires further improvement.

### 3.5. Compound Fungicide T10 Demonstrated Significantly Superior Control Efficacy Against Root Rot Caused by A2, A3, and A6 Compared to the Single Formulations of T1, T2, and T9

**Figure 8:**
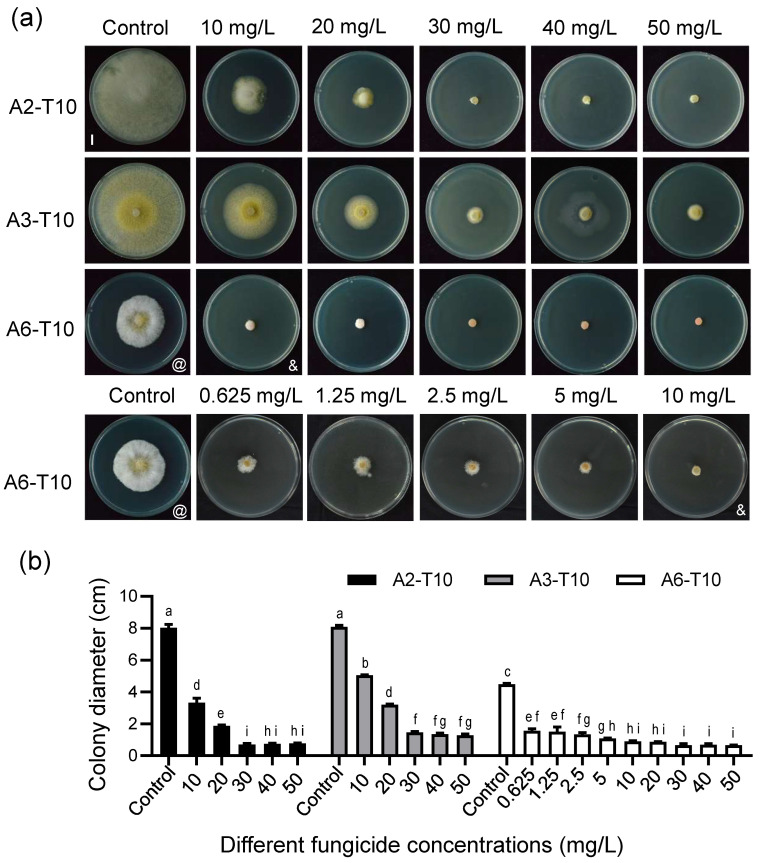
Effects of different concentrations of T10 compound fungicide on the growth rate of pathogenic strains A2, A3, and A6 for *C. violifolia* root rot. (**a**) Colony growth of strains A2, A3, and A6 after 5 days of incubation on medium containing different concentrations of T10 fungicide. Colonies grown on fungicide-free medium served as the negative control. Scale bar = 1 cm. Symbols @ and & indicate results for the same strain under the same treatment condition. (**b**) Statistical results of colony diameter (including the original inoculum plug diameter of 6 mm) for pathogenic strains A2, A3, and A6 after 5 days of incubation on medium containing different concentrations of T10 compound fungicide. Error bars indicate the SD of three plates in each experiment. Different letters in (**b**) represent significant differences at *p* < 0.05 using one-way ANOVA with Tukey’s multiple comparisons test.

Given the suboptimal inhibitory effects of single fungicides against pathogens A2 and A3, a compound formulation was developed by combining fungicides with inhibition rates above 30% against all three pathogens (A2, A3, and A6). The resulting compound fungicide T10 exhibited inhibition rates of 97.80%, 90.95%, and 98.40% against A2, A3, and A6 at 50 mg/L, respectively, with EC_50_ values of 7.3130 mg/L, 12.2983 mg/L, and 0.1781 mg/L. These represent reductions of 66.0%, 77.7%, and 92.1% compared to the most effective single application of propiconazole (T9) (Table 2 and Table S7). Notably, at 10 mg/L, T10 increased inhibition rates by 62.62%, 77.53%, and 20.85% compared to T9 alone. The toxicity of the resulting compound fungicide T10 against the three pathogens was evaluated, with results showing inhibition rates of 97.80%, 90.95%, and 98.40% against A2, A3, and A6 at 50 mg/L, respectively (Table S7). EC_50_ values of 7.3130 mg/L, 12.2983 mg/L, and 0.1781 mg/L were obtained, representing reductions of 66.0%, 77.7%, and 92.1% compared to the most effective single application of propiconazole (T9) (Table 2). When applied at a low concentration of 10 mg/L, the inhibition rates against the three pathogens increased by 62.62%, 77.53%, and 20.85%, respectively, compared to the single application of propiconazole (T9). Moreover, synergism analysis revealed potent synergistic interactions among the three components of T10. For strain A6, the observed EC_50_ (EC_50_(ob), 0.1781 mg/L) was substantially lower than the theoretical value (EC_50_(th), 4.4475 mg/L), resulting in an SR of 24.97 (Table S8). This value far exceeds Wadley’s synergism threshold (SR > 1.5), confirming strong synergistic effects and demonstrating markedly superior efficacy compared to individual fungicide treatments (Figure 8; Tables S7 and S8).

**Figure 9:**
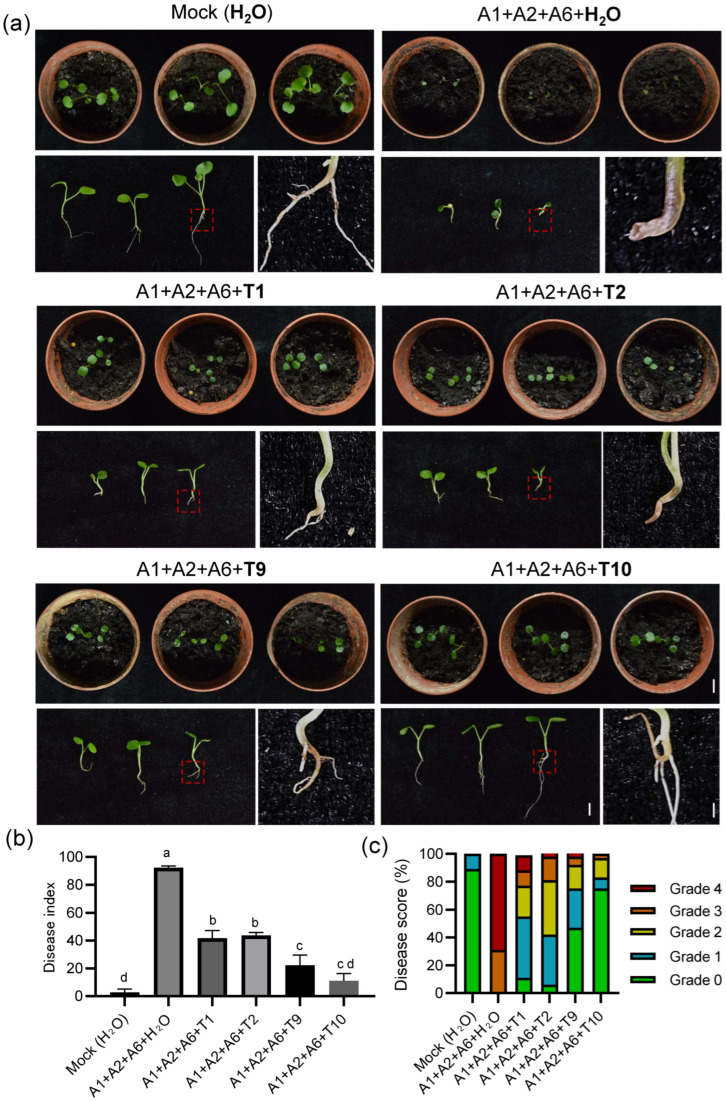
The compound fungicide T10 demonstrated significantly superior control efficacy against root rot caused by A2, A3, and A6 compared to the single formulations of T1, T2, and T9. (**a**) Control efficacy against root rot following application of compound fungicide T10 and single formulations of T1, T2, and T9 after simultaneous inoculation with pathogenic strains A2, A3, and A6. Seedlings were cultivated in soil for 7 days and then exposed to root rot pathogens A2, A3, and A6 (10 mL each, 1 × 10^6^ CFU/mL) for 3 days. Mock inoculation was performed with deionized water. Treatment with deionized water after inoculation with pathogens A2, A3, and A6 served as the negative control. Red dashed boxes indicate the region magnified on the right-hand side. Scale bar = 1 cm. (**b**,**c**) Disease index (**b**) and disease score (**c**) of various treatments in (**a**). Disease symptoms in *C. violifolia* were evaluated 7 days after fungicide application. Error bars represent the SD of data collected from at least 36 plants within each treatment group. Different letters in (**b**) represent significant differences at *p* < 0.05 using one-way ANOVA with Tukey’s multiple comparisons test.

To further validate the control efficacy of compound fungicide T10 against root rot in *C. violifolia*, seedlings were inoculated simultaneously with A2, A3, and A6 pathogens for 3 days, followed by application of compound fungicide T10 and single formulations of T1, T2, and T9. Disease symptoms were observed and recorded 7 days post-treatment. The results showed that plants mock-inoculated with *C. violifolia* grew normally; by contrast, negative control plants inoculated with the three pathogens but without fungicide application exhibited severe root rot symptoms, including root necrosis and leaf wilting. Application of either T1, T2, or T9 single formulations or T10 compound fungicide significantly alleviated root rot symptoms, with notably superior efficacy observed for the T10 compound compared to the single formulations (Figure 9a). The disease index (Figure 9b) and disease score (Figure 9c) results were consistent with the observed phenotypes, confirming that compound fungicide T10 demonstrated significantly superior control efficacy against root rot caused by A2, A3, and A6 compared to the single formulations of T1, T2, and T9.

**Figure 10:**
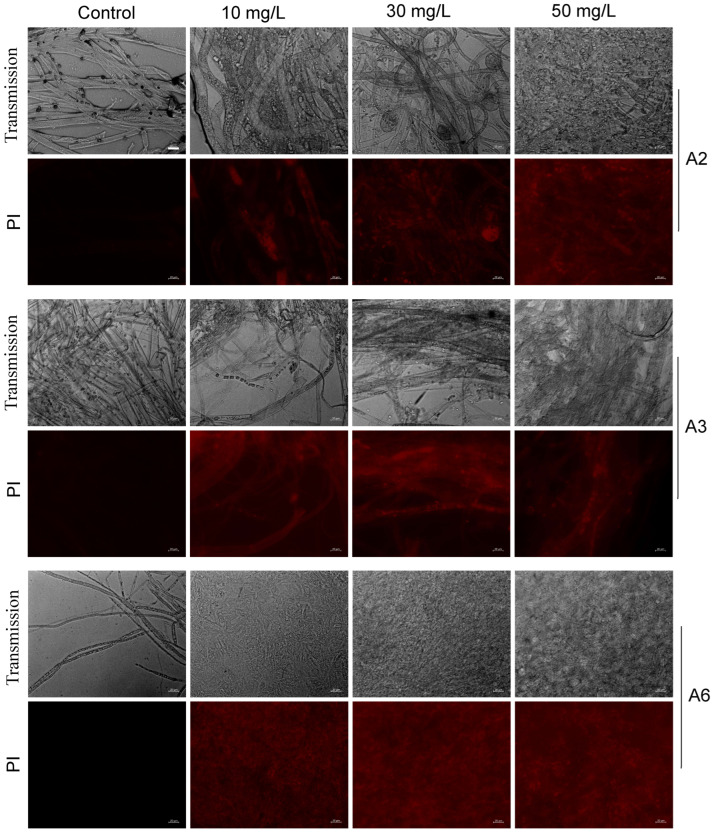
Effects of different concentrations of T10 compound fungicide on plasma membrane damage for pathogenic strains A2, A3, and A6. The strains were treated with various concentrations of T10 fungicide, stained with PI solution, and observed under a fluorescence microscope. Fluorescence intensity reflects the degree of membrane damage. Strains treated with PBS alone served as negative controls. Scale bar = 20 µm.

The plasma membrane serves as a critical site for material exchange between the interior and exterior of cells, protecting internal cellular structures and functions [39]. PI permeates damaged or dead cells, intercalates into double-stranded DNA, and emits red fluorescence, the intensity of which is reflected in membrane damage. However, it fails to affect intact plasma membranes [38]. After treatment of A2, A3, and A6 strains with different concentrations of T10 fungicide, the pathogen mycelia exhibited extensive red fluorescent areas, whereas untreated mycelia showed no red fluorescence. Moreover, the red fluorescent area increased as fungicide concentration increased, indicating that the fungicide exerted a significant disruptive effect on the plasma membrane integrity of the pathogens (Figure 10). These findings are consistent with T10-induced membrane damage as a potential contributor to its antifungal effect, though additional mechanistic studies are needed to confirm this relationship.

## 4. Discussion

### 4.1. Main Pathogens of C. violifolia Root Rot and Their Pathogenic Potential

*Cardamine violifolia* is a selenium-enriched cruciferous plant with high nutritional value, containing substantial amounts of organic selenium and nervonic acid. It has promising potential for applications in neuroprotection and intervention of neurodegenerative diseases [33]. However, with the expansion of artificial cultivation, root rot has emerged as one of the primary obstacles limiting its large-scale production. So, root rot not only threatens the planting safety of *C. violifolia* but also affects the production of its high-value components, including organic selenium and nervonic acid, thereby compromising the economic and medicinal value of this functional vegetable crop. Similar to other cruciferous crops, this disease is typically caused by co-infection of multiple soilborne fungi, including *Fusarium*, *Mucor*, and *Aspergillus* spp. as a complex pathogenic consortium [13,40]. These fungi can produce various extracellular degrading enzymes and mycotoxins that disrupt root cell wall and membrane structures, leading to tissue necrosis, impaired nutrient absorption, plant wilting, and death [41]. Due to the strong genetic diversity of these pathogens, cryptic infection strategies, and long-term survival in soil, conventional control measures are often ineffective, and reliance on single fungicides frequently results in inconsistent control efficacy and development of resistance [13].

For the first time, this study identified three major pathogens causing *C. violifolia* root rot in Enshi: *Aspergillus costaricensis*, *Mucor circinelloides* cf. *lusitanicus*, and *Fusarium pernambucanum* (Figure 2, Figure 4 and Figure 5; Table S2). All three induced typical browning and tissue necrosis in host roots, with *F. pernambucanum* exhibiting the strongest pathogenicity (Figure 4 and Figure 5). Previous research has reported that *F. pernambucanum* is a significant pathogen causing papaya root rot in northeastern Brazil [42], which supports its high pathogenic potential in plant infection. Additionally, *M. circinelloides* f. *lusitanicus* has been confirmed to cause postharvest mucor rot in citrus fruits in California, representing a saprophytic–parasitic fungus with established pathogenic capacity [43]. In contrast, *A. costaricensis* has not been reported as a plant pathogen; instead, strain LS18 of this species has been found to efficiently produce xylanase, rapidly hydrolyzing xylan from agricultural waste (e.g., *Lycium barbarum* leaves) into fermentable sugars, demonstrating significant potential for biomass degradation and bioenergy applications [44]. Although these three pathogens differ in pathogenicity, this study revealed a typical co-infection phenomenon (Figure 9). Related studies indicate that weak pathogens can facilitate infection and spread of aggressive pathogens by altering the rhizosphere microenvironment or secreting secondary metabolites, creating synergistic infection effects [13]. This complex pathogenic system significantly increases the complexity of root rot, making it difficult for single-target fungicides to achieve long-term, stable control efficacy.

### 4.2. Synergistic Effects of the Novel Compound Fungicide T10

Among the eight commonly used fungicides screened, propiconazole exhibited the lowest EC_50_ values and strongest inhibitory activity against all three pathogens (Figure 6 and Figure 7; Table 1). This is primarily attributed to propiconazole being a demethylation inhibitor (DMI) in the triazole class. It blocks ergosterol biosynthesis in pathogen cell membranes, leading to structural abnormalities, increased permeability, and inhibited hyphal growth [45,46]. This class of fungicides is characterized by broad-spectrum activity, high efficacy, and long residual effects. It has been widely used to control fungal diseases caused by *Fusarium* spp., *Rhizoctonia solani*, and other pathogens. Despite these advantages, resistance to DMI fungicides has been reported in various pathogens, including *Colletotrichum*, *Venturia*, *Fusarium*, *Monilinia*, and *Zymoseptoria tritici* [47,48]. Therefore, relying solely on propiconazole is insufficient for comprehensive control of complex root rot. Rational fungicide mixing is considered an effective strategy to reduce pathogen resistance, improve control efficacy, and lower costs [49,50,51]. Cross-resistance analysis indicates that propiconazole has resistance overlap with prochloraz, but not with other agents, demonstrating its potential for rotation and formulation [52].

To overcome the limitations of single-agent use, this study selected hymexazol and difenoconazole combined with propiconazole to develop the novel compound fungicide T10. In vitro toxicity assays demonstrated that the EC_50_ values of T10 are significantly lower than those of propiconazole alone. At a low dose of 10 mg/L, the inhibition rates against pathogens A2, A3, and A6 increased by 62.62%, 77.53%, and 20.85%, respectively, compared to the single agent, showing significant synergistic effects (Figure 8; Table 2 and Table S8). Furthermore, soil drench experiments provided phenotypic observations of root rot symptoms and statistical results for DI values, which demonstrated that the compound fungicide T10 provided better control of root rot caused by the three mixed pathogens (Figure 9).

The ternary compound fungicide T10 contains two DMI fungicides: T9 (propiconazole) and T2 (difenoconazole). DMI fungicides typically possess a single site of action, making it easy for pathogens to develop resistance through target gene mutations [47,53]. Moreover, positive cross-resistance exists between propiconazole and difenoconazole, meaning that resistance to one often leads to failure of the other [47,48]. Therefore, during formulation design, we incorporated T1 fungicide hymexazol, which has a non-DMI mode of action (inhibiting DNA/RNA synthesis), to diversify the targets and mitigate resistance risk. Additionally, we recommend avoiding sublethal doses during application to ensure effective pathogen killing, as well as regularly monitoring pathogen sensitivity in the field to effectively manage the resistance risk associated with fungicide use.

### 4.3. Molecular Mechanism of the Novel Compound Fungicide T10 in Controlling Root Rot

Hymexazol is a broad-spectrum fungicide used to control root rot, particularly soil-borne diseases caused by *Fusarium* spp., *Pythium* spp., and *Aphanomyces* spp. in rice (*Oryza sativa*) or vegetables [54]. Difenoconazole is a sterol biosynthesis inhibitor that can cause cell membrane damage by interfering with sterol biosynthesis [55]. When combined, they could enhance efficacy by promoting the penetration and accumulation of propiconazole in hyphae [21,56]. PI fluorescence staining results suggested that T10 is associated with membrane integrity disruption in treated pathogens, as evidenced by increased cell membrane permeability (Figure 10). These observations are consistent with membrane damage as a potential component of the antifungal mechanism, though further studies are required to confirm its precise role. Moreover, the inhibition rates against the three pathogens increased by 20–70% compared to propiconazole alone, showing clear positive synergistic effects (Tables S7 and S8). Therefore, T10 provides more stable and efficient control efficacy in complex root rot management through multi-pathway, multi-target combined antifungal action.

Traditional root rot research has mostly focused on single pathogens. By contrast, this study emphasizes the importance of multi-pathogen co-infection, aligning with recent theories on the systematic control of plant disease [57]. Future research should combine analysis of fungicide translocation characteristics and pathogen signaling pathways to further elucidate the molecular mechanisms of compound formulations. Additionally, it is necessary to combine T10 with biocontrol agents to form a “chemical–biological bidirectional regulation” system [57], achieving safer, more efficient, and sustainable management of root rot.

## 5. Conclusions

This study systematically revealed the composition of the main pathogenic fungi causing *C. violifolia* root rot through tissue isolation, morphological observation, and molecular identification. All three pathogens induced typical root browning, tissue necrosis, and growth inhibition, showing clear characteristics of co-infection. Through fungicide screening, propiconazole was found to have inhibitory effects on all three pathogens, but its efficacy as a single agent was limited. To improve efficacy, this study combined propiconazole (T9), hymexazol (T1), and difenoconazole (T2) to develop the novel ternary compound fungicide T10. Toxicity assays and soil control efficacy demonstrated that T10 at a low concentration of 10 mg/L significantly increased inhibition rates against the three pathogens compared to single agents, showing significant synergistic effects. Moreover, PI fluorescence staining further revealed that T10 can significantly disrupt pathogen cell membrane structure, causing abnormal increases in membrane permeability, thereby achieving stable, broad-spectrum, and highly efficient control efficacy. These findings provide a robust scientific foundation for effective disease management and sustainable production of this high-value nutritional crop.

## Figures and Tables

**Table 1:** Virulence regression equations and EC_50_ values for mycelial growth inhibition of pathogenic strains A2, A3, and A6 for *C. violifolia* root rot using T1, T2, and T9 fungicide treatments.

Treatments with Different Fungicides	Virulence Regression Equation	EC_50_ for Inhibition (mg/L)	Correlation Coefficient (r)
A2-T9	y = 0.7047x − 0.4314	21.5214	0.8458
A3-T9	y = 0.4892x − 0.379	55.0963	0.9541
A6-T9	y = 0.4183x + 0.3521	2.2504	0.9909
A6-T1	y = 0.3233x + 0.2616	5.7328	0.8153
A6-T2	y = 0.8662x − 0.5925	17.9445	0.9660

y, inhibition rate. x, logarithm of fungicide concentration.critical values for df = 3 (n = 5): r_0.05_ = 0.878, r_0.01_ = 0.959.

**Table 2:** Toxicity regression equations for mycelial growth inhibition of pathogenic strains A2, A3, and A6 for *C. violifolia* root rot using T10 compound fungicide treatments.

Treatments	Virulence Regression Equation	EC_50_ for Inhibition (mg/L)	Correlation Coefficient (r)
A2-T10	y = 0.5282x + 0.1341	7.3130	0.9047
A3-T10	y = 0.7718x − 0.3417	12.2983	0.9395
A6-T10	y = 0.1897x + 0.7435	0.1781	0.8547

y, inhibition rate. x, logarithm of fungicide concentration. critical values for df = 3 (n = 5): r_0.05_ = 0.878, r_0.01_ = 0.959.

## Data Availability

The data presented in this study are available on request from the corresponding author. The data are not publicly available due to privacy reasons.

## Supplementary Materials

The following supporting information can be downloaded at: https://www.mdpi.com/article/10.3390/biology15040368/s1. Table S1. Strains and accession numbers used for the construction of the rDNA ITS and RPB2 phylogenetic trees; Table S2. Comparison of homology in rDNA-ITS sequences cloned from *C. violifolia* root rot isolates with GenBank reference sequences; Figure S1. Comparison of colony morphology between the original isolates and re-isolated pathogens; Table S3. Names, codes, and concentration of fungicides; Table S4. Inhibition rates of different fungicides on the growth of the A6 pathogen causing *C. violifolia* root rot; Table S5. Inhibition rates of T9 fungicide on the growth of A2, A3, and A6 pathogens causing *C. violifolia* root rot; Table S6. Inhibition rates of T1 and T2 fungicides on the growth of the A6 pathogen causing *C. violifolia* root rot; Table S7. Inhibition rates of T10 compound fungicides on the growth of A2, A3, and A6 pathogens causing *C. violifolia* root rot; Table S8. Synergistic interaction analysis of the ternary compound fungicide T10 against pathogenic strains A6 causing root rot in *C. violifolia* using Wadley’s method.
