# Supplementary material for: Isolation, Identification, and Management Strategies for the Root Rot Pathogen of Cardamine violifolia
Source: Biology (Basel). 2026 Feb 22;15(4):368. doi: 10.3390/biology15040368 (PMC12938698; doi:10.3390/biology15040368)
Supplement: Supplementary file 1 [file biology-15-00368-s001.zip › biology-4127542-supplementary.pdf]

Supplementary Data

Table S1. Strains and accession numbers used for the construction of the rDNA ITS and RPB2 phylogenetic trees

Table S2. Comparison of homology in rDNA-ITS sequences cloned from *C. violifolia* root rot isolates with GenBank reference sequences

Figure S1. Comparison of colony morphology between the original isolates and re-isolated pathogens

Table S3. Names, codes, and concentration of fungicides

Table S4. Inhibition rates of different fungicides on the growth of the A6 pathogen causing *C. violifolia* root rot

Table S5. Inhibition rates of T9 fungicide on the growth of A2, A3, and A6 pathogens causing *C. violifolia* root rot

Table S6. Inhibition rates of T1 and T2 fungicides on the growth of the A6 pathogen causing *C. violifolia* root rot

Table S7. Inhibition rates of T1 and T2 fungicides on the growth of the A6 pathogen causing *C. violifolia* root rot

Table S8. Synergistic interaction analysis of the ternary compound fungicide T10 against pathogenic strains A6 causing root rot in *C. violifolia* using Wadley's method

Table S1. Strains and accession numbers used for the construction of the rDNA ITS and RPB2 phylogenetic trees

Strain name	Strain codes	Gene fragment codes	
		rDNA-ITS	RPB2
Mucor atramentarius	CBS 202.28	PP882861	MF495246
Mucor circinelloides cf. *lusitanicus*	CBS 108.17	JF439685	JN993501
Mucor variicolumellatus	CBS 236.35	JN205979	-
Mucor irregularis	CBS 103.93	MH862387	JX976279
Mucor racemosus	CBS 111228	JN205991	-
Mucor bainieri	CBS 293.63	NR_103628	-
Mucor ramosissimus	CBS 135.65	NR_103627	MF495253
Mucor velutinosus	MYA-4766	NR_111682	XM_064826394
Aspergillus tubingensis	CBS 559.65	MH858714	LC707883
Aspergillus costaricensis	CBS 115574	NR_103604	HE984361
Aspergillus piperis	CBS 112811	NR_077191	MK450798
Aspergillus welwitschiae	CBS 139.54	NR_137513	MN969100
Aspergillus awamori	CBS 139.52	MH856964	HE984360
Aspergillus luchuensis	CBS 205.80	LC884988	MN969081
Aspergillus foetidus	CBS 121.28	NR_163668	-
Aspergillus niger	ATCC 16888	NR_111348	MW659710
Aspergillus pulverulentus	CBS 558.65	EU821317	HE984368
Fusarium hainanense	CGMCC3.19478	NR_164597	JX171635
Fusarium arcuatisporum	CGMCC3.3.19493	NR_164593	MW474507
Fusarium luffae	CGMCC3.3.19497	NR_164594	OR037271
Fusarium equiseti	NRRL 26419	NR_121457	PV580250
Fusarium ipomoeae	CGMCC3.3.19496	NR_164596	PV053561
Fusarium irregulare	CGMCC3.3.19489	NR_164595	JX171645
Fusarium pernambucanum	URM 7599	NR_163754	PV866757
Fusarium humuli	CGMCC3.3.19374	NR_164598	OR257587
Fusarium incarnatum	NRRL 32867	GQ505705	MZ604830
Fusarium chlamydosporum	CBS 145.25	NR_172283	OR425371
Fusarium oxysporum	CBS 132473	MH866031	MT568980
Thanatephorus cucumeris	CBS 280.36	MH855798	MF804914
Ellisomyces anomalus	CBS 243.57	NR_145284	-
Methanobacterium alcaliphilum	DSM 3459	AB496639	-

Symbol - indicates no corresponding sequence was retrieved.

Table S2. Comparison of homology in rDNA-ITS sequences cloned from *C. violifolia* root rot isolates with GenBank reference sequences

Strain ID	rDNA-ITS length	Homologous strains	Coverage	Similarity	Strain GenBank accession numbers
A1	862 bp	*Mucor circinelloides* cf. *lusitanicus*	100%	98.03%	CBS 108.17
A2	869 bp	*Aspergillus costaricensis*	100%	96.55%	CBS 115574
A3	862 bp	*Mucor circinelloides* cf. *lusitanicus*	100%	99.88%	CBS 108.17
A4	864 bp	*Fusarium pernambucanum*	100%	99.54%	URM 7599
A5	863 bp	*Fusarium pernambucanum*	100%	96.54%	URM 7599
A6	865 bp	*Fusarium pernambucanum*	100%	96.54%	URM 7599
A7	853 bp	*Fusarium luffae*	98%	95.87%	CGMCC3.3.19497
A8	608 bp	*Mucor circinelloides* cf. *lusitanicus*	100%	99.54%	CBS 108.17
A9	607 bp	*Mucor circinelloides* cf. *lusitanicus*	99%	99.88%	CBS 108.17

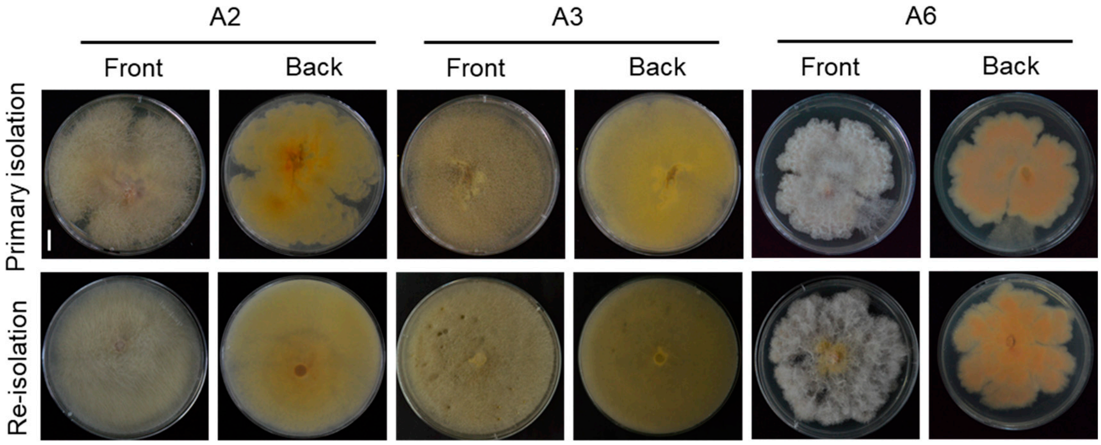

Figure S1. Comparison of colony morphology between the original isolates and re-isolated pathogens. Colony morphology after 12 days of incubation. Front, front view; Back, back view. Scale bar = 1 cm.

Table S3. Names, codes, and concentration of fungicides

Codes	Names	Concentration	Forms	Manufacturers
T1	Hymexazol	97%	Technical material (TM)	Shanghai Macklin Biochemical Co., Ltd
T2	Difenoconazole	95%	Technical material (TM)	Shanghai Macklin Biochemical Co., Ltd.
T3	Prochloraz	450 g/L	Emulsion in Water (EW)	Shanghai Hulian Biological Pharmaceutical Co., Ltd.
T4	Metalaxyl·mancozeb	58%	Wettable Powder (WP)	Qingdao Hansheng Biotechnology Co., Ltd
T5	Chlorothalonil	40%	Suspension Concentrate (SC)	Shanghai Hulian Biological Pharmaceutical Co., Ltd.
T6	Carbendazim	98%	Technical material (TM)	Shandong Keyuan Biochemical Co., Ltd.
T8	Kasugamycin·oxine-copper	36%	Suspension Concentrate (SC)	Hebei Jindelun Biochemical Technology Co., Ltd.
T9	Propiconazole	50%	Microemulsion (ME)	Shandong Qingdao Gelisi Pharmaceutical Co., Ltd.

Table S4. Inhibition rates of different fungicides on the growth of the A6 pathogen causing *C. violifolia* root rot

Fungicide	Measured value (cm)			Mean colony growth diameter (cm)	Inhibition rate (%)
T1	2.40	2.60	2.10	2.37 ± 0.21d	38%
T2	0.50	1.20	1.00	0.90 ± 0.29e	77%
T3	2.80	2.90	3.20	2.97 ± 0.17cd	23%
T4	3.30	3.50	3.90	3.57 ± 0.25bc	7%
T5	2.90	3.10	3.00	3.00 ± 0.08bd	22%
T6	3.30	3.50	3.90	3.57 ± 0.25bc	7%
T8	5.90	6.70	6.70	6.43 ± 0.38a	-68%
T9	0.21	0.23	0.22	0.23 ± 0.01f	94%
Control	3.50	3.90	4.10	3.83 ± 0.25b	-

Colonies grown on fungicide-free medium served as the negative control. Different letters represent significant differences at $P < 0.05$ using one-way ANOVA with Tukey's multiple comparisons test. Symbol - indicates exclusion from the calculation.

Table S5. Inhibition rates of T9 fungicide on the growth of A2, A3, and A6 pathogens causing *C. violifolia* root rot

T9 Concentration (mg/L)	A2		A3		A6	
	Mean colony growth diameter (cm)	Inhibition rate (%)	Mean colony growth diameter (cm)	Inhibition rate (%)	Mean colony growth diameter (cm)	Inhibition rate (%)
0.625	-	-	-	-	1.97 ± 0.25ab	27.16%
1.25	-	-	-	-	1.70 ± 0.08b	37.04%
2.5	-	-	-	-	1.27 ± 0.12bc	53.09%
5	-	-	-	-	0.90 ± 0.08c	66.67%
10	6.21 ± 0.15b	19.90%	6.59 ± 0.24a	9.13%	0.67 ± 0.26d	75.31%
20	2.93 ± 0.22c	62.22%	5.09 ± 0.37b	29.81%	0.23 ± 0.05e	92.04%
30	2.94 ± 0.05c	62.11%	4.93 ± 0.04b	32.03%	0.27 ± 0.09e	90.00%
40	2.75 ± 0.26c	64.51%	4.15 ± 0.15c	42.74%	0.23 ± 0.12e	92.04%
50	1.98 ± 0.18d	74.42%	4.12 ± 0.11c	43.11%	0.23 ± 0.12e	92.04%
Control	7.75 ± 0.11a	-	7.25 ± 0.17a	-	2.70 ± 0.42a	-

Colonies grown on fungicide-free medium served as the negative control. Different letters represent significant differences at $P < 0.05$ using one-way ANOVA with Tukey's multiple comparisons test. Symbol - indicates exclusion from the calculation.

Table S6. Inhibition rates of T1 and T2 fungicides on the growth of the A6 pathogen causing *C. violifolia* root rot

T1 Concentration (mg/L)）	A6		T2 Concentration (mg/L)	A6	
	Mean colony growth diameter (cm)	Inhibition rate (%)		Mean colony growth diameter (cm	Inhibition rate (%)
10	1.99 ± 0.23b	54.40%	10	3.11 ± 0.15b	28.80%
20	1.11 ± 0.07c	74.62%	20	2.02 ± 0.28c	53.66%
30	1.05 ± 0.14c	75.91%	30	1.68 ± 0.05c	61.53%
40	1.02 ± 0.06c	76.64%	40	0.67 ± 0.08d	84.72%
50	0.96 ± 0.07c	78.11%	50	0.51 ± 0.11d	88.24%
Control	4.37 ± 0.40a	-	Control	4.37 ± 0.40a	-

Colonies grown on fungicide-free medium served as the negative control. Different letters represent significant differences at $P < 0.05$ using one-way ANOVA with Tukey's multiple comparisons test. Symbol - indicates exclusion from the calculation.

Table S7. Inhibition rates of T10 compound fungicides on the growth of A2, A3, and A6 pathogens causing *C. violifolia* root rot

T10 Concentration (mg/L)	A2		A3		A6	
	Mean colony growth diameter (cm)	Inhibition rate (%)	Mean colony growth diameter (cm)	Inhibition rate (%)	Mean colony growth diameter (cm)	Inhibition rate (%)
0.625	-	-	-	-	0.97 ± 0.10bc	75.06%
1.25	-	-	-	-	1.13 ± 0.36b	70.69%
2.5	-	-	-	-	0.74 ± 0.09bcd	80.95%
5	-	-	-	-	0.48 ± 0.03ce	87.62%
10	2.72 ± 0.22b	63.42%	4.44 ± 0.03b	40.66%	0.29 ± 0.04de	92.16%
20	1.26 ± 0.05c	83.04%	2.59 ± 0.04c	65.42%	0.27 ± 0.02de	92.82%
30	0.10 ± 0.06d	98.65%	0.86 ± 0.05d	88.45%	0.06 ± 0.08e	98.41%
40	0.14 ± 0.03d	98.07%	0.75 ± 0.06de	90.03%	0.08 ± 0.06e	97.79%
50	0.16 ± 0.03d	97.80%	0.68 ± 0.06e	90.95%	0.06 ± 0.02e	98.40%
Control	7.43 ± 0.17a	-	7.48 ± 0.08a	-	3.74 ± 0.20a	-

Colonies grown on fungicide-free medium served as the negative control. Different letters represent significant differences at $P < 0.05$ using one-way ANOVA with Tukey's multiple comparisons test. Symbol - indicates exclusion from the calculation.

Table S8. Synergistic interaction analysis of the ternary compound fungicide T10 against pathogenic strains A6 causing root rot in *C. violifolia* using Wadley's method

Treatment	EC 50(ob)/ (mg/mL)	EC50(th)/(mg/mL)	Synergistic ratio (SR)
A6-T10	0.1781	4.4475	24.9722

Synergism was assessed by Wadley's method: SR = EC_{50}(th)/EC_{50}(ob), where EC_{50}(th) = (a+b+c)/[a/EC_{50}(A)+b/EC_{50}(B)+c/EC_{50}(C)]. SR > 1.5, 0.5–1.5, and < 0.5 indicate synergism, additivity, and antagonism, respectively.
